# The Preparation of Cyclic Binary Block Polymer Using Bimolecular Homodifunctional Coupling Reaction and Characterization of Its Performance as a Drug Carrier

**DOI:** 10.3390/molecules30030599

**Published:** 2025-01-29

**Authors:** Guiying Kang, Muxin Lu, Kang Zhou, Cuiyun Yu, Hua Wei

**Affiliations:** 1 College of Chemical engineering and Technology, Tianshui Normal University, Tianshui 741001, China; 18193117856@163.com; 2 China PetroChina Lanzhou Lubricating Oil R & D Institute, Lanzhou 730060, China; zhoukang_rhy@petrochina.com.cn; 3 Hunan Province Cooperative Innovation Center for Molecular Target New Drug Study, Department of Pharmacy and Pharmacology, University of South China, Hengyang 421001, China; yucuiyunusc@hotmail.com

**Keywords:** cyclic binary block polymer, bimolecular homodifunctional coupling reaction, drug carrier, performance

## Abstract

There is relatively little research on cyclic amphiphilic block polymers, having both hydrophilic and hydrophobic segments placed in the ring and thus resulting in a higher degree of topological restriction, as drug vehicles. Cyclic amphiphilic binary block polymer is synthesized by the click coupling reaction of bimolecular homodifunctional precursors. The results indicate that cyclization between linear polymer precursors is successful if the trace linear by-products generated are ignored, which also suggests that the small molecule bifunctional terminating agent applied in traditional bimolecular homodifunctional ring-closure process can be extended to large molecule. Moreover, the study on the self-assembly behavior of polymers shows that, compared with linear counterparts, the stability and drug loading capacity of micelles based on the resultant cyclic polymer are not significantly improved due to the influence of topological structure and linear impurities. Nevertheless, drug loaded micelles formed by the obtained cyclic polymers still exhibit superior cellular uptake ability. It can be seen that topological effects do play an irreplaceable role in the application performance of polymers. Therefore, the construction and synthesis of cyclic and its derivative polymers with moderate topological confinement and high purity may be a key direction for future exploration of polymer drug delivery carriers.

## 1. Introduction

Cyclic polymers without chain ends have a range of unique physical properties, such as reduced hydrodynamic radius [1,2,3], resulting from their constrained conformations; higher glass transition temperature [4,5,6] (*T*_g_) and increased crystallization and melting temperature [7,8,9] (*T*_c_, *T*_m_), which arise from the changes in the mobility and interactions (inter- and intrachain) of a polymer segment, and lower melt viscosity [10,11,12,13], deriving from their reduced chain entanglements, which are ascribed to their different behaviours in solutions, bulk, and melts. These intrinsic properties mentioned above foster differences in the self-assembly behavior of cyclic polymer with respect to linear ones. For example, self-assembled micelles formed by cyclic polymers exhibit greatly enhanced stability [14,15,16], relative to both temperature and ionic strength, which has been confirmed by the raised cloud point temperature and elevated salting-out concentrations. In addition, cyclic polymers display a two-stage degradation behaviour with topological dependence [17,18], possibly enhancing micelle-based drug delivery efficiency. Meanwhile, the structure-dependent behaviour of the enhanced permeability and retention (EPR) effect [19,20] and prolonged blood circulation times [21,22,23] have also been observed when cyclic polymers serve as biomedical material. These distinctive application properties have successfully caught the attention of chemists, who have also made unremitting efforts in the synthesis methods, elucidation of topological effects, and potential applications of cyclic polymers. To this day, although challenges still exist in purification and large-scale production, two mature approaches for synthesizing cyclic polymers are provided, including ring-closure and ring-expansion strategies [24,25,26]. The ring-closure technology is further divided into three classes: bimolecular homodifunctional, unimolecular homodifunctional, and unimolecular heterodifunctional method. There are different advantages and limitations, as well as scope of applicability for each approach. Take the ring-closure approach as an example, especially for the bimolecular homodifunctional type, it is only suitable for cyclization of relatively low-molecular-weights polymers with a small difunctional linker under high-dilution conditions. Moreover, this traditional ring-closure method is commonly used for anionic polymerization of vinyl monomers; for instance, H. Höcker [27] prepared macrocyclic polystyrene using sodium naphthalene as an initiator, which generates a bifunctional “living” chain and α,α’-dichloro-p-xylene as a bifunctional terminating agent. G. Hild et al. [28] chose potassium naphthenide and dibromo-p-xylene as a telechelic initiator and bifunctional coupling agent, respectively, to produce ring-shaped polystyrene at extremely low concentrations. Given that the bimolecular ring-closure technique inevitably contains the competition between forming cyclic polymers and extended linear ones, the formation of line-cycle mixed polymers is a definite result. However, there are few reports on how the existence of a small amount of linear contaminants in cyclic polymers affects its self-assembly behavior and the performance of its self-assemblies as a drug carrier.

In order to investigate (1) the feasibility of hydrophobic polymers as macromolecular linkers in the process of bimolecular homodifunctional cyclization and (2) the impact of side reactions on the performances of the final product as a drug carrier, here, using the copper-catalyzed azide–alkyne cycloaddition (CuAAC) developed by Laurent and Grayson [29] to couple the hydrophilic chain poly (ethylene glycol) (PEG), terminated with two azide groups, and hydrophobic chain poly (ε-caprolactone) (PCL), with two alkyne end groups, an attempt is made to synthesize cyclic binary block polymer poly (ethylene glycol)-*b*-poly (ε-caprolactone) (*c*(PEG-*b*-PCL)). The influences of trace linear contaminates on the self-assembly behavior of the target polymers and the in vitro anti-tumor performance of its self-assemblies as drug carriers are studied via the size and critical micelle concentration (CMC) values of self-assembled micelles and the drug-loading capacity of the micelles, as well as drug release behavior and cytotoxicity of drug-loaded micelles. Simultaneously, the results are compared with the linear analogs having the same composition and similar molecular weight. This study may provide additional evidence suggesting that the application properties of polymers can be greatly affected by the topological structure.

## 2. Results

### 2.1. Synthesis and Characterization of Cyclic Binary Block Polymer c(PEG-b-PCL) and Linear Analog PEG-b-PCL

**Scheme 1:**
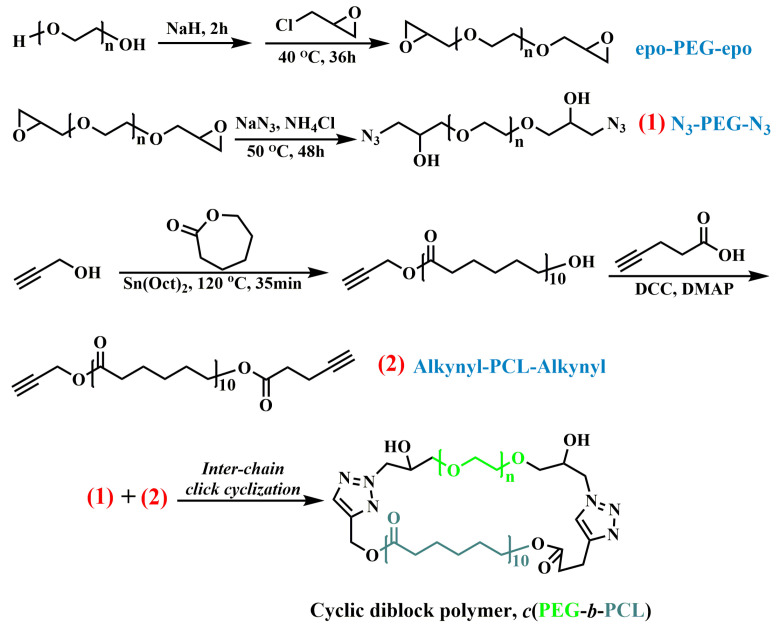
Synthesis of cyclic binary block polymer c(PEG-b-PCL).

The synthesis of target products *c*(PEG-*b*-PCL) by coupling bimolecular homodifunctional precursors using the CuAAC method primarily involves the preparation of bifunctional hydrophilic and hydrophobic precursors, N_3_-PEG-N_3_ and Alkynyl-PCL-Alkynyl (Scheme 1).

**Figure 1:**
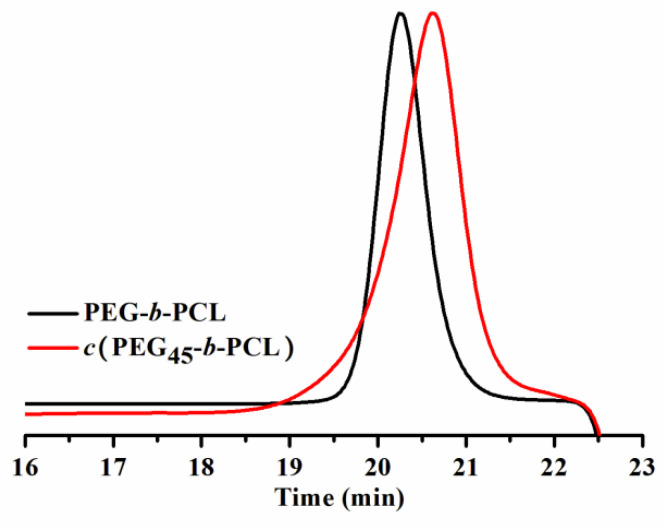
SEC elution traces of the linear analogue PEG-*b*-PCL and cyclic diblock polymer *c*(PEG-*b*-PCL) using DMF as an eluent.

A hydrophilic segmen, N_3_-PEG-N_3_, is prepared through the terminal conjugation between the OH-PEG-OH and the reactive epoxy groups (epo-PEG-epo), followed by a ring-opening reaction of the epoxy group with sodium azide. The end-capping efficiency of -epo [30] is absolute and is obtained by calculating the ratio of the integrated intensity of a characteristic signal of the epoxide ring at 3.17 ppm (3) to that of the methylene protons of PEG at 3.78 ppm (1^/^) in the ^1^H NMR spectrum of epo-PEG-epo (Figure S1). With the implementation of the ring-opening reaction, the specific signals of the epoxide ring (peak 3 and 4 in Figure S1) are completely replaced by a new peak at 3.34 ppm (3), which is assigned to the methine proton of -CHOH in the ^1^H NMR spectrum of N_3_-PEG-N_3_ (Figure S2), besides the evidence of characteristic absorption of azide groups in FT-IR spectrum (Figure S3), which confirms the successful acquisition of a bifunctional hydrophilic polymer. The hydrophobic chain Alkynyl-PCL-Alkynyl is obtained by a combination of ring-opening polymerization (ROP) of ε-CL with propargyl alcohol as an initiator and an esterification reaction of 4-pentynoic acid under DCC and DMAP catalysis. The ^1^H NMR (Figure S4) results indicate that ROP is successful, and the degree of polymerization (DP) of PCL is 10 and is calculated from the integrated-area ratio of the methylene proton on the initiator (peak 2) and the methylene proton near the hydroxyl group on PCL (peak 6). The success of the esterification reaction is also verified by the characteristic peak of the pentynyl appearing on ^1^H NMR (Figure S5), and the substitution rate of the alkynyl group is determined to be 100% by calculating the integral-area ratio of peaks 2 and 8. Subsequently, the target cyclic diblock polymer *c*(PEG-*b*-PCL) is synthesized via coupling of N_3_-PEG-N_3_ and Alkynyl-PCL-Alkynyl in highly diluted deoxygenated DMF containing catalytic CuBr/PMDETA. Published studies [31] have shown that the cyclization efficiency of homodifunctional bimolecular coupling ring-closure reactions exceeds 90% under highly diluted conditions with a concentration of less than 10^−5^ M. Thus, we choose a cyclization reaction concentration of 5 × 10^−5^ M in our experiment, hoping to achieve approximately equal reaction efficiency. The characteristic peaks of methylene proton both on PEG and PCL simultaneously appeared in the ^1^H NMR (Figure S6) of the final product, indicating that bimolecular homodifunctional precursors are effectively coupled. Most importantly, the SEC (Figure 1) efflux curve of the cyclic polymer shifts almost entirely to the right, which represents the direction of decreasing fluid dynamics volume, further declaring that bimolecular coupling cyclization is successful performed and has a higher cyclization efficiency in highly diluted environments; although, there is a tailing phenomenon on the high-molecular-weight side that is ascribed to unavoidable competitive reactions or deactivation of functional group at one end [25,32], resulting in a cyclization rate of not 100%.

The synthesis of linear counterpart PEG-*b*-PCL is similar to that of the cyclic polymers, except that the end groups of both linear precursors only carry one active functional group, namely one azide and alkyne group (Scheme S1). The successful production of PEG-N_3_, PCL-Alkynyl, and target polymer PEG-*b*-PCL has also been confirmed by ^1^H NMR (Figures S4, S7, S8 and S10), FT-IR (Figure S9), and SEC (Figure S11). The molecular weight (MW) and polydispersity index (PDI) of all synthesized polymers are concluded in Table S1.

### 2.2. Characterization of Self-Assembled Micelles

The characterization of micelles formed by cyclic and linear polymers consists of primarily three aspects, including (i) the thermodynamic stability, (ii) the hydrodynamic diameter and size distribution, and (iii) the morphology of micelles. The critical micelle concentrations (CMCs) that reflect the stability of self-assembled micelles are determined to be 0.012 and 0.023 g/L for cyclic and linear polymers (Figure S12), respectively, by the pyrene probe method. Although the CMC value of cyclic polymers is as low as half that of linear counterparts, the degree of decrease is inconspicuous, which may be due to the presence of trace linear structures affecting the stacking behavior of the cyclic polymer. These results suggest a slightly higher thermodynamic stability of cyclic-polymer-based micelles than that of linear analogs, which may lead to a longer blood circulation time for the drug carrier formed by cyclic polymers since the smaller the CMC, the less likely the carrier is to disintegrate in highly diluted body fluid environments [33].

**Figure 2:**
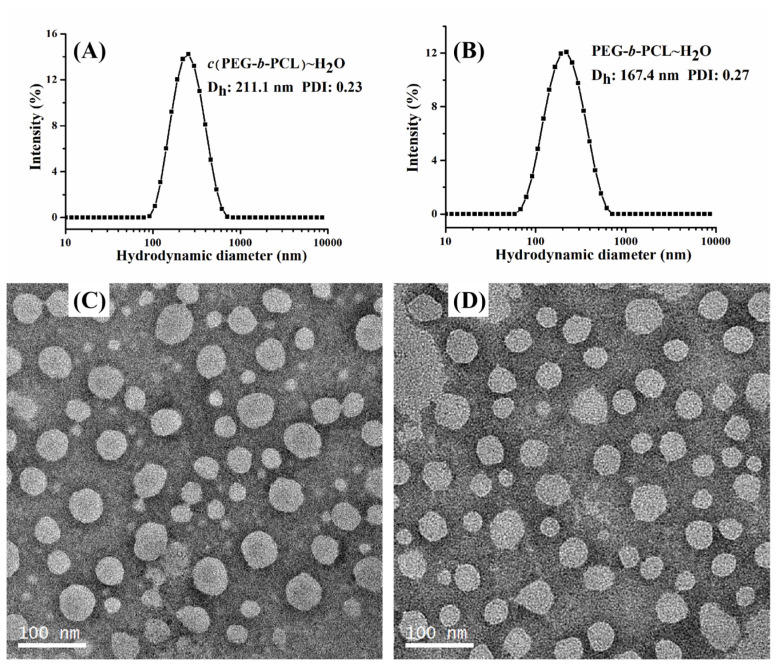
Hydrodynamic diameter and size distribution and TEM images of the micelles self-assembled from cyclic (**A**,**C**) and linear (**B**,**D**) polymers at a polymer concentration of 0.3 mg/mL.

The hydrodynamic diameter (*D*_h_) and size distribution (PDI) of micelle solution with a concentration of approximately 0.3 mg/mL (greater than CMC) obtained through dialysis are investigated using dynamic light scattering (DLS), and the results show that the *D*_h_ is 211.1 nm and 167.4 nm, and the PDI is 0.23 and 0.27 for micelles self-assembled by cyclic and linear topological polymers (Figure 2A and Figure 2B), respectively. There are two possible reasons why the *D*_h_ of the cyclic polymers is slightly larger than that of linear counterparts: one is macro-ring steric hindrance effect [34,35], that is, almost no interference from trace linear contaminates in the products with ring-polymers as the main component, which can definitely affect the packing behavior during the self-assembly process of polymers, and the other is restrictive chain collapse [36], which is caused by hydrophobic segments placed in the ring in the process of self-assembly, making it difficult to form a structurally dense hydrophobic core. A TEM of the samples reveals that the morphology of micelles based on both polymers exhibits a spherical shape (Figure 2C,D), demonstrating that the cyclic topology structure does not affect the morphology of the self-assemblies from this binary block polymer. However, the results from a computer simulation indicate that the self-assemblies morphology of the mixture mainly consisting of cyclic polymers is a mixed state [37], containing sphere, cylinder, lamella, and vesicle, and is mainly composed of a mixture of lamella and vesicle. But, here, only one micelle shape, namely spherical, is obtained. The possible reason for this difference in shapes is that the simulation conditions are set, that is, controllable, such as fixed mixing ratio, hydrophobicity, and the ratio of hydrophilic–hydrophobic, while the actual experimental process is complex and uncontrollable in advance, particularly in terms of the uniformity of the polymer structure. In addition, the sizes acquired from the TEM are approximately 55 nm and 40 nm, respectively, for micelles formed by cyclic and linear topological polymers, which are consistent with the result of DLS that polymers with ring topology have a larger size but are much smaller than those gained from DLS as for specific values because of the different sample states corresponding to the two tests; that is, the dry state of TEM is relative to the hydrated state of DLS sample [38].

### 2.3. In Vitro Drug Loading and Drug Release Study

The model drug Doxorubicin (DOX), a hydrophobic antineoplastic agent with red fluorescence, is encapsulated into the micellar core via the hydrophobic interactions in the dialysis. The drug-loading capacity (DLC) and entrapment efficacy (EE) of DOX-loaded micelles self-assembled by cyclic and linear polymers are calculated to be 4.32% and 35.12% and 4.02% and 30.14%, respectively, based on the standard curve of DOX in the corresponding solution. This almost identical result is far inferior to previously reported DLC and EE increase in micelles because of the stronger hydrophobic interactions [35,39] and higher colloidal stability [40,41] for cyclic topology, which is probably attributed to the interference of linear impurities in the drug-packaging process and the mismatch of hydrophilic and hydrophobic segments ratios.

**Figure 3:**
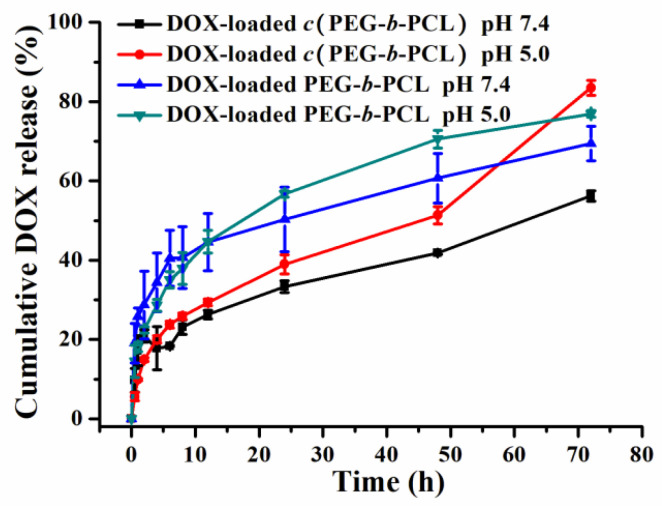
In vitro drug release profiles of *c*(PEG-*b*-PCL)@DOX and PEG-*b*-PCL@DOX micelles under different conditions at 37 °C.

In vitro drug release results performed in the normal physiological pH of 7.4 and intracellular endosomal/lysosomal pH of 5.0 at 37 °C suggest that the DOX release amounts of drug-loading micelles cultured for 72 h are 56% and 69% at pH 7.4 and 83% and 77% at pH 5.0 (Figure 3), respectively, for cyclic polymers and linear counterparts. From this, although the stability of cyclic polymer micelles is greatly decreased due to the presence of trace linear contaminates, it still has higher stability than that of linear analogs in physiological environments, which is more conducive to the long-term circulation of the drug delivery system in the body and to reduce the side effects of free DOX to normal tissues in theory [42]. In addition, in acidic conditions, the release of DOX from the micellar core is accelerated, ascribed to the increased solubility of DOX as the protonation of the amino group of DOX. However, as for this cyclic polymer *c*(PEG-*b*-PCL) with a determined hydrophilic/hydrophobic ratio, it is not entirely suitable as a drug carrier because the release amount of DOX relying on it has exceeded 50% under physiological conditions, hinting that it cannot achieve the stability of clinical application as a drug delivery system. Considering the poor stability of linear polymers with the same composition and molecular weight, it is speculated that the reason for the above results may be related to both the purity of the cyclization product and the ratio of hydrophilicity–hydrophobicity balance. The composition, topological structure, and concentration of polymers can definitely affect the morphology, size, and stability of its self-assemblies based on the reported studies [43,44]. From a thermodynamic point of view [37,45], the key factor affecting them is the conformational entropy of polymer chains, which is related to the mean-square radius of gyration (*S*^2^) of hydrophobic blocks and can be changed by the three conditions mentioned above. It can be understood that if the system is a cyclic polymer completely free of linear impurities, *S*^2^ remains unchanged due to topological limitations, and the system is in a constant state of entropic penalty, but, if it is a mixture, *S*^2^ is no longer fixed under the influence of trace linear structures, and the entropy of the system has a nonmonotonic tendency, which will cause dynamic micro-changes in the morphology and size of aggregates, leading to a decrease in their stability. This also explains the fact from a deeper level that the topology of polymers has a direct impact on its self-assembly behavior and its self-assemblies properties, because the difference between the research and comparison objects here lies only in the structure.

### 2.4. In Vitro Cellular Uptake and Cytotoxicity Studies

**Figure 4:**
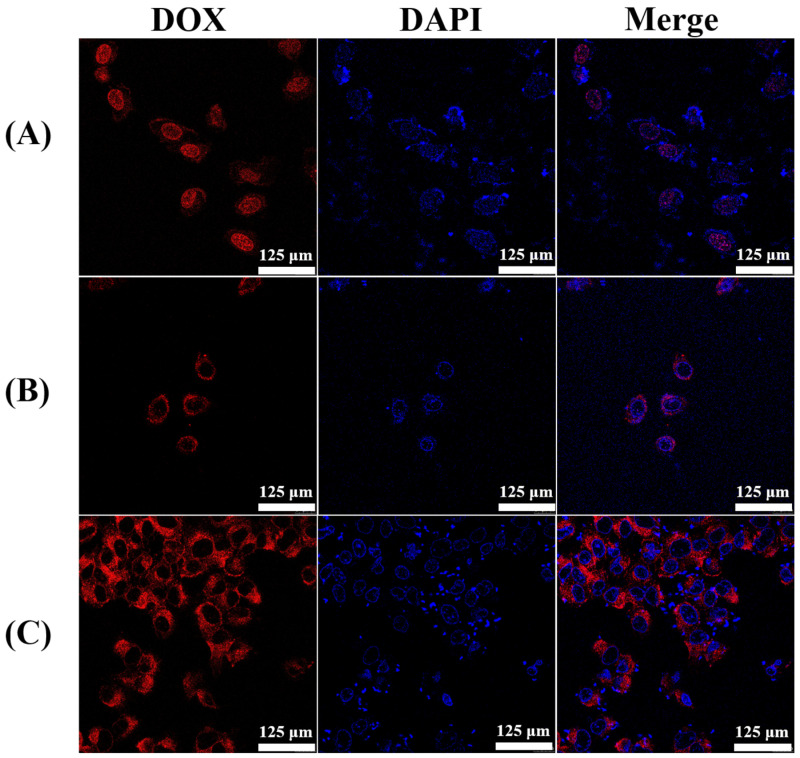
Confocal imaging of free DOX (**A**), micelles of PEG-*b*-PCL@DOX (**B**), and micelles of *c*(PEG-*b*-PCL)@DOX (**C**) uptake in HeLa cells (nuclei stained blue with DAPI). Note that cells were treated with polymer or free drug at 50% of their respective IC_50_ values to minimize cell death.

For a drug carrier, being able to be successfully and effectively absorbed by cells is one of its important qualities. Here, the cellular uptake efficiency is qualitatively evaluated through the confocal imaging in the HeLa cells incubated with free DOX, *c*(PEG-*b*-PCL)@DOX, and PEG-*b*-PCL@DOX micelles for 4 h. The strong red fluorescence of DOX observed in the cytoplasm and/or nuclei proves that the DOX can be successfully delivered by both formulations to the cells and underwent intracellular trafficking in a pattern similar to free DOX (Figure 4). Compared with linear PEG-*b*-PCL@DOX, the *c*(PEG-*b*-PCL)@DOX micelles have a larger area of the red, implying that they arevmore easily absorbed into cells.

**Figure 5:**
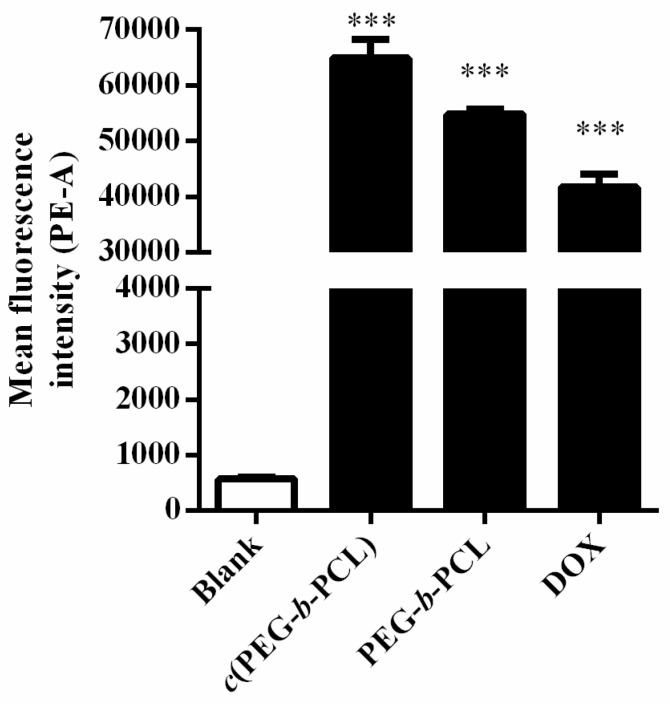
Quantitative measurements of the mean fluorescence intensity after incubation with free DOX, PEG-*b*-PCL@DOX, and *c*(PEG-*b*-PCL)@DOX in the HeLa cells via flow cytometry (4 h of incubation, DOX concentration = 49 μg/mL, and 10,000 cells counted). The data were expressed as mean ± SD, n = 3, *** *p* < 0.005.

To quantify the cellular uptake efficiency of both micelle formulations, including the same DOX content (49 μg/mL), acflow cytometry (FCM) analysis is executed in the HeLa cells after co-culture, and the obtained order of mean fluorescence intensity from low to high is free DOX, PEG-*b*-PCL@DOX, and *c*(PEG-*b*-PCL)@DOX micelles (Figure 5). These results are in good agreement with the confocal observation. The lowest intensity of free DOX may be related to the lower phagocytosis caused by the elimination of p-glycoprotein during its diffusion through the cell membrane [46], while the highest intensity of *c*(PEG-*b*-PCL)@DOX micelles may be influenced by the higher DLC and the faster drug release at pH 5.0 revealed by the in vitro drug loading and drug release study of them.

Good biocompatibility is a necessary condition for carrier applications, and the evaluation results of the in vitro cytotoxicity of various micelle constructs in the HeLa cells by the MTS cell viability assay have confirmed that both blank micelles of *c*(PEG-*b*-PCL) and PEG-*b*-PCL possess this condition, since the cell viability still exceeds 90% even at a polymer concentration of up to 200 μg/mL after 24 h of incubation (Figure S13). Furthermore, the half-maximal inhibitory concentrations (IC_50_) of free DOX, *c*(PEG-*b*-PCL)@DOX, and PEG-*b*-PCL@DOX micelles are obtained as 1.36 (1.24–1.48) μg/mL, 74.57 (56.47–98.47) μg/mL, and 98.97 (76.33–128.3) μg/mL (Figure S14), respectively. Both micelle formulations have lower cytotoxicity than that of free DOX, which possibly originated from the differences in the internalization mechanisms and drug release kinetics of the drug-loaded micelles. Additionally, compared to PEG-*b*-PCL@DOX, the reduced IC_50_ of the *c*(PEG-*b*-PCL)@DOX micelles is likely a result of its higher drug release.

### 2.5. In Vitro Degradation Study

The impact of the topological effects on the degradation behavior of the cyclic amphiphilic diblock polymer is mainly reflected in the slowing down of the degradation rate caused by the transition of its topological structure from ring to line during the degradation process, which has been confirmed by Grayson group [18]. Because the degradation of polymer carriers directly affects their drug release and drug delivery efficiency, the degradation study of cyclic and linear diblock polymers is conducted based on the identical experimental procedure reported [30]. From the SEC characterization of degradation products at various time periods within 24 h (Figure S15), it can be seen that the retention time (RT) of the linear counterpart has significantly shifted towards the low-molecular-weight side after only 6 h of degradation, which is attributed to the formation of small molecule substances generated from the breakage of ester bonds on the linear polymer chains. On the contrary, the RT of the cyclic polymers decreases from 20.63 to 20.57 min after 24 h of degradation, indicating an increase in the molecular weight of degradation products. The reason for this result is that the breakage of ester bonds on the cyclic chain forms a linear diblock polymer with a larger effective molecular weight. Reviewing the relevant results of drug release, we find that the cumulative release of cyclic *c*(PEG-*b*-PCL)@DOX micelles after 24 h is 33.4% at pH 7.4 and 39.0% at pH 5.0, while the linear control group is 50.3% and 56.7%, respectively. This difference in data may also be related to the unique two-stage degradation behavior guided by cyclic topology, as it is more favorable to protecting drugs from leakage due to carrier disintegration, which also helps to improve drug delivery efficiency evidenced by the cellular absorption results of its drug-loaded micelles.

## 3. Conclusions

The amphiphilic cyclic binary block polymer *c*(PEG-*b*-PCL) is tentatively synthesized by a bimolecular homodifunctional coupling reaction, demonstrating the feasibility of hydrophobic polymers PCL as macromolecular homodifunctional linkers, but the structural characterization of the final product confirms that the cyclization efficiency of this method is less than 100% even under extremely dilute conditions due to the presence of competitive reactions forming linear byproducts. In products mainly composed of cyclic diblock polymers *c*(PEG-*b*-PCL), the self-assembly behavior of the polymer exhibits topological guidance behavior, such as relatively larger hydrodynamic diameter (211.1 nm), lower CMC value (0.012 g/L), and higher drug-loading capacity and entrapment efficacy (DLC and EE, 4.32% and 35.12%) of self-assembled micelles. However, the appearance of trace linear by-products does indeed affect the self-assembly behavior of the resultant polymer and the performance of the self-assemblies as a drug carrier, making the excellent performance that cyclic polymers should exhibit less apparent, for instance, their improvement in the stability and drug-loading capacity of micelles is not significant compared to their linear counterpart (CMC is 0.23 g/L, DLC and EE are 4.02% and 30.14%) with the same composition and molecular weight. In spite of this, the results of the in vitro cellular uptake still show that the cyclic polymers are superior to the linear analogue in drug delivery. In addition, the cyclic polymers exhibit a topology-directed two-stage degradation behavior. Depending on the above research results, advanced topological structures, and cyclic binary diblock polymers is beneficial for improving the application properties of drug delivery systems established by amphiphilic polymers. But it must be emphasized that continuous efforts should be implemented in synthesis methods, large-scale preparation, regulation of hydrophilic and hydrophobic ratios, and expansion of block composition, which is crucial for the development of high-performance clinical drug delivery carriers in the future.

## Data Availability

The data that support the findings of this study are available from the corresponding author upon reasonable request.

## Supplementary Materials

The following supporting information can be downloaded at: https://www.mdpi.com/article/10.3390/molecules30030599/s1, Figure S1: ^1^H NMR spectrum of epo-PEG-epo in CDCl_3_; Figure S2: ^1^H NMR spectrum of N_3_-PEG-N_3_ in CDCl_3_; Figure S3: FT-IR spectrum of the N_3_-PEG-N_3_; Figure S4: ^1^H NMR spectrum of Alkynyl-PCL in CDCl_3_; Figure S5: ^1^H NMR spectrum of Alkynyl-PCL-Alkynyl in CDCl_3_; Figure S6: ^1^H NMR spectrum of *c*(PEG-*b*-PCL) in CDCl_3_; Figure S7: ^1^H NMR spectrum of PEG-Br in CDCl_3_; Figure S8: ^1^H NMR spectrum of PEG-N_3_ in CDCl_3_; Figure S9: FT-IR spectrum of the PEG-N_3_; Figure S10: ^1^H NMR spectrum of PEG-*b*-PCL in CDCl_3_; Figure S11: SEC elution traces of the PEG-Br, PEG-N3, PCL-Alkynyl, and linear analogue PEG-b-PCL using DMF as an eluent; Figure S12: (A&C) Fluorescence emission spectra of pyrene at different concentrations of *c*(PEG-*b*-PCL) and PEG-*b*-PCL, (B&D) Plots of fluorescence intensity of *I*_373_ and *I*_384_ as function of logarithm of concentrations of *c*(PEG-*b*-PCL) and PEG-*b*-PCL; Figure S13: In vitro cytotoxicity of blank *c*(PEG-*b*-PCL) (A) and PEG-*b*-PCL micelles (B) in HeLa cells. Cell viability was determined by MTS assay and expressed as % viability compared to the untreated cells control. The data were expressed as mean ± SD, n = 3; Figure S14: In vitro cytotoxicity of *c*(PEG-*b*-PCL)@DOX (A) and PEG-*b*-PCL@DOX (B) micelles and free DOX (C) in HeLa cells for 24 h of incubation. The data were expressed as mean ± SD, n = 3; Figure S15: SEC analyses of the degraded products of linear (A) and cyclic (B) polymers at various degradation times; Table S1: Summary of MW and PDI of all synthesized polymers; Scheme S1: Synthesis of linear analogue PEG-*b*-PCL.
