# Supplementary material for: The Preparation of Cyclic Binary Block Polymer Using Bimolecular Homodifunctional Coupling Reaction and Characterization of Its Performance as a Drug Carrier
Source: Molecules. 2025 Jan 29;30(3):599. doi: 10.3390/molecules30030599 (PMC11820105; doi:10.3390/molecules30030599)
Supplement: Supplementary file 1 [file molecules-30-00599-s001.zip › molecules-3435328-supplementary.pdf]

Supporting Information

List of Contents (19 pages, 15 figures, 1 scheme, 1 table)

1. Experimental section

2. Figure S1-S15, Scheme S1, Table S1

Experimental section

Materials

Monomethoxy Poly(ethylene glycol) (PEG, M_n=2000 g/mol), Poly(ethylene glycol) (PEG, M_n=2000 g/mol), Stannous (II) octanoate ($Sn(Oct)_2$), Copper(I) bromide (CuBr, >99.999%), 2-Bromoisobutyryl bromide (α-iBuBr), and Doxorubicin hydrochloride (DOX·HCl, ≥98%) were purchased from Sigma-Aldrich and used as received. Epichlorohydrin (Aladdin, 99.7%), *N,N′*-dicyclohexylcarbodiimide (DCC, J&K, 98%), 4-dimethylamino pyridine (DMAP, J&K, 99%), 4-Pentynoic acid (J&K, 95%), *N,N,N′,N′′,N′′*-pentamethyldiethylenetriamine (PMDETA, Aladdin, 99%), and Triethylamine (TEA, Aladdin) were used as received. Sodium azide (NaN_3) and Ammonium chloride (NH_4Cl) were obtained from Sinopharm Chemical ReagentCo., Ltd (China) without further purification before use. Propargyl alcohol (Tianjin Chemical Reagent Factory, China) was refluxed with K_2CO_3 and distilled under reduced pressure before use. ε-Caprolactone (ε-CL, J&K, 99%) was dried over CaH_2 and distilled under reduced pressure prior to use. Dichloromethane (CH_2Cl_2, DCM), toluene (refluxed with Na and distilled under reduced pressure before use), tetrahydrofuran (THF), *N,N*-dimethylformamide (DMF), anhydrous ether, and anhydrous methanol were provided from Lianlong Bohua (Tianjin, China) Pharmaceutical Chemistry Co., Ltd.

Instruments and Measurements

^{1}H NMR spectra were recorded on a JNM-ECS 400 MHz spectrometer (JEOL, Tokyo, Japan) operated in the Fourier transform mode using deuterated chloroform($CDCl_3$) and tetramethylsilane (TMS) as the solvents and internal reference, respectively. The molecular weight (MW) and the polydispersity index (PDI=M_w/M_n) of all of the synthesized polymers were determined by the size exclusion chromatography and multiangle laser light scattering (SEC-MALLS) at 60 °C using HPLC-grade DMF containing 0.1 wt % LiBr as the eluent of SEC at a flow rate of 1 mL/min. Tosoh TSK-GEL R-3000 and R-4000 columns (Tosoh Bioscience) were connected in series to a Agilent 1260 series (Agilent Technologies), an interferometric refractometer (Optilab-rEX, Wyatt Technology), and a MALLS device (DAWN EOS, Wyatt Technology). The MALLS detector was operated at a laser wavelength of 690.0 nm. The FT-IR spectroscopic measurements were conducted on a NEXUS 670 FT-IR spectrometer (Nicolet, WI, USA) and solid samples were pressed into potassium bromide (KBr) pellet prior to the measurements.

Synthesis of epoxide-terminated PEG (epo-PEG-epo)

The synthesis of epo-PEG-epo was similar to the reported paper[1]. The PEG (2.5 mmol) was dissolved in 150 mL of toluene to remove the water from PEG by azeotropic

distillation with toluene. After leaving about 50 mL of toluene in the reaction flask, it was cooled to room temperature under nitrogen protection, and then NaH was added and activated for 2 h. Next, epichlorohydrin was added dropwise to the reaction solution and the mixture was stirred at 40 °C for 48 h. The product was washed with distilled water, followed by drying over anhydrous magnesium sulfate. After filtration, the solution was concentrated and precipitated into cold ether. (yield 87%)

Synthesis of N_3-PEG-N_3

N_3-PEG-N_3 was prepared by the ring-opening reaction of epo-PEG-epo with NaN_3. The epo-PEG-epo (0.01 mmol) was dissolved in 50 mL of DMF, and then NH_4Cl (0.2 mmol) and NaN_3 (0.2 mmol) were added, and the reaction was performed at 50 °C for 48 h. At the end of the reaction, the product was diluted with 200 mL of CH_2Cl_2, and extracted with water for three times, and then dried with anhydrous $MgSO_4$. After filtration, the most of the solvent was removed by rotary evaporate, followed by precipitation into cold diethyl ether. The N_3-PEG-N_3 was collected by drying under vacuum (yield 86%).

Synthesis of Alkynyl-PCL-Alkynyl

The synthesis of Alkynyl-PCL-Alkynyl was divided into two steps. Firstly, the preparation of Alkynyl-PCL was carried out by ROP of ε-CL using propargyl alcohol as the initiator. Briefly, ε-CL (100 mmol), propargyl alcohol (20 mmol) and $Sn(Oct)_2$ (0.2 mmol) served as catalyst were placed in 10 mL Schlenk flask with a magnetic stirring bar. The reaction mixture was degassed through three pump-freeze-thaw cycles and then immersed in a thermo-stated oil bath at 120 °C for 27 min. The reaction was quenched with liquid nitrogen, diluted with THF, and then precipitated into ice methanol. The product was dried under vacuum to obtain a white solid Alkynyl-PCL (yield 80%).

Next, the terminal hydroxyl group of Alkynyl-PCL was absolutely converted to alkynyl function by an esterification reaction. Simply, Alkynyl-PCL (0.84 mmol), DCC (3.34 mmol) and DMAP (0.33 mmol) were dissolved with dried toluene and charged in a round-bottom flask. 4-Pentynoic acid (2.51 mmol) was added after the above solution cooled to 0 °C and the reaction was maintained 30 min at the temperature. After that, the reaction mixture was stirred at room temperature for 24 h. After completed, the insoluble *N*, *N'*-dicyclohexylurea (DCU) solids were removed by filtration, and then the organic solution was precipitated into cold methanol to obtain the final product Alkynyl-PCL-Alkynyl (yield 75%).

Synthesis of *c*(PEG-*b*-PCL)

The amphiphilic cyclic binary block polymer *c*(PEG-*b*-PCL) was prepared by bimolecular homodifunctional coupling reaction. Specifically, 800 mL of DMF was added to a 1000 mL three-neck flask which was degassed by bubbling nitrogen flow for 1 h, and the temperature slowly rises to 100 °C. 49-Fold molar equivalent of PMDETA and CuBr were added to the flask under the protection of nitrogen flow. The hydrophilic N_3-PEG-N_3 (0.04 mmol) and hydrophobic Alkynyl-PCL-Alkynyl (0.04 mmol) chains were dissolved in DMF (10 mL). After deoxygen for 40 minutes, the polymer solution was injected into the above DMF solution containing copper catalyst thermostated at 100 °C via a syringe pump at a fixed rate of 0.2 mL/h. After complete injection of the polymer solution, the reaction was proceeded for another 24 hours at 100 °C. After completed, the reaction solution was cooled to room temperature and DMF was removed under reduced pressure. The concentrated residue was re-dissolved directly with CH_2Cl_2 and extracted with saturated EDTA solution to eliminate any excess copper. Organic phase was dried over anhydrous $MgSO_4$,

and the solution was concentrated and precipitated into cold ether. The target polymer, *c*(PEG-*b*-PCL), was harvested after dried under vacuum (yield 58%).

Synthesis of linear analogue PEG-*b*-PCL

Firstly, three linear precursors, PEG-Br, PEG-N_3 and Alkynyl-PCL, were prepared. The PEG (Monomethoxy, 5 mmol) was dissolved in 100 mL of anhydrous DCM, and then it was cooled to 0 °C in an ice bath, and TEA (25 mmol) was added into it. Later, α-iBuBr (20 mmol) was added dropwise into the above solution, and the reaction was continued for 40 minutes at 0 °C. Remove the ice bath, the reaction was continued for another 12 h when the temperature returns to room. After reacted, the reaction solution was washed with HCl (1M), NaOH (1M) and saturated NaCl solution in turn, and subsequently dried with anhydrous $MgSO_4$. Then, the solution was concentrated, and the residue was re-dissolved by $CHCl_3$ and further separated with alkaline alumina columns to remove excessive amine. The products of column separation were precipitated into ice ether, and the white solid precursor PEG-Br was obtained by vacuum drying (yield 91%).

The 1 mmol of PEG-Br and 20 mmol NaN_3 were dissolved into 10 mL of DMF, and reaction was continued for 48 h at 45 °C. After completed, the insoluble salt was eliminated by washing with distilled water and the target polymer PEG-N_3 was collected by precipitation of residue products from DCM to ether (yield 83%).

The preparation of Alkynyl-PCL is the same as described in the synthesis of Alkynyl-PCL-Alkynyl.

The linear analogue PEG-*b*-PCL was prepared by click coupling of PEG-N_3 and Alkynyl-PCL. Namely, PEG-N_3 (0.14 mmol) and Alkynyl-PCL (0.12 mmol) were dissolved with 4 mL DMF and subsequently charged in a 25 mL Schlenk flask equipped with a magnetic stirring bar. The reaction mixture was degassed through three pump-freeze-thaw cycles to remove any trace of oxygen in the system. Subsequently, CuBr (0.14 mmol) catalyst and PMDETA (0.14 mmol) were added quickly under a nitrogen flow. After another three freeze-pump-thaw cycles, the Schlenk flask was sealed and placed in a thermostated oil bath at 45 °C to start the polymerization. After 48 h, the reaction mixture was diluted with DMF, and transferred directly to a dialysis tube (MWCO: 3.5 kDa) and dialyzed against distilled water to remove the copper catalyst. The linear polymer, PEG-*b*-PCL, was obtained by freeze-drying (yield 69%).

Preparation and characterization of the self-assembled micelles

Linear or cyclic polymer (1 mg) was dissolved in 1 mL of DMF. The polymer solution was transferred to a dialysis tube and dialyzed against distilled water for 24 h to obtain a micelle solution with a concentration of approximately 0.3 mg/mL.

TEM images were recorded on a JNM-2010 instrument operating at an acceleration voltage of 200 keV. TEM samples were prepared by dropping 4 μL of micelle solution obtained by dialysis onto a carbon-coated copper grid. After deposition for 20 min, the excess solution was removed by a strip of filter paper. The sample was further stained with phosphotungstic acid (1 % w/w) and dried in air prior to visualization.

The average hydrodynamic size of the self-assembled micelles was measured by dynamic light scattering (DLS) on a Zeta sizer (Nano ZS, Malvern, Worcestershire, UK) at a fixed detection angle of 173°. The sample solution was passed through a Millipore 0.45 μm pore-sized syringe filter prior to measurements. Polymer solution with a concentration of 0.3 mg/ml was evaluated.

Critical Micelle Concentration was measured using pyrene as a fluorescence probe and fluorescence spectra were recorded on a LS55 luminescence spectrometer (Perkin-Elmer). 80 μL of Pyrene solution (3×10^{-6} M in acetone) was added to containers, and the

acetone was allowed to evaporate. Then 4 mL of polymer aqueous solution at different concentrations were added to the containers containing the pyrene residue and the combined solution of pyrene and copolymers was equilibrated at room temperature in dark for 24 h prior to measurements. The final concentration of pyrene was 6×10^{-8} M in water. Excitation was carried out at 340 nm, and emission spectra were recorded ranging from 350 to 600 nm. Excitation and emission bandwidths were 5 nm and 2.5 nm, respectively. From the pyrene emission spectra, the intensities (peak height) of I_{373} and I_{384} were recorded, and the CMC value was determined from the intersection of the tangent to the curve at the inflection with the horizontal tangent to the curve through the points at low concentration[2].

In vitro drug loading and drug release

DOX·HCl (2 mg) and TEA (96 μL) were dissolved in 4 mL of DMF and stirred overnight in dark at room temperature to obtain DOX base. Next, the polymer (20 mg) dissolved in 2 mL of DMF was added to the above DOX solution and stirred at room temperature for 1 h. The above mixture was later added dropwise into 6 ml of ultra-purified water under vigorous stirring. After stirring for another 1 h, the solution was transferred to a dialysis tube and dialyzed against 5 L of distilled water for 24 h, which was renewed every 3 h during the course of initial 12 h to remove any unencapsulated free DOX. Finally, the drug-loaded micelles were harvested by freeze-drying. To determine the drug loading content (DLC) and entrapment efficiency (EE), the freeze-dried drug-loaded micelles were re-dispersed in PBS (pH 7.4). The concentration of DOX was determined by measuring the absorbance at 499 nm using a Lambda 35 UV-Vis spectrometer (Perkin-Elmer). The EE and DLC were calculated through the following equations,

$$\text{DLC (\%)} = W_{\text{drug loaded in particles}} / W_{\text{particles}} \times 100\% \quad (1)$$

$$\text{EE (\%)} = W_{\text{drug loaded in particles}} / W_{\text{drug fed for encapsulation}} \times 100\% \quad (2)$$

The in vitro drug release study was investigated in PBS (pH 7.4,150 mM) and saline sodium citrate (SSC, pH 5.0, 150 mM) at 37 °C, respectively. The drug-loaded micelles were re-dispersed in PBS buffer, and the concentration of its is 0.5 mg/mL. 1 mL of the solution was loaded in the dialysis bag (MWCO: 3.5 kDa), which was immersed in a tube loaded with 25 mL of the release medium. The tube was placed in a horizontal laboratory shaker thermostated with a constant temperature of 37 °C and a stirring speed of 120 rpm. At the predetermined time intervals, 3 mL of the release medium in tube was taken out and equal volume of fresh medium was added. The drug concentration was calculated by measuring the absorbance at 499 nm according to a standard calibration curve. The experiment was performed in quadruplicate for each sample.

Confocal imaging

Confocal imaging was carried out according to the following steps. Briefly, HeLa cells were seeded in 12-well plates at a plating density of 1×10^3 cells per well in 1 mL of complete growth medium and incubated in a 37 °C, 5% CO_2 environment for 24 h. Solutions of DOX-loaded micelles were prepared in complete growth medium at concentrations equal to 50% of their IC_{50} value and then added to the wells and incubated for 4 h at 37 °C. Cells were later rinsed with PBS and fixed with 4% paraformaldehyde (PFA) solution for 20 min at room temperature. Finally, cells were counterstained with 2-(4-amidinophenyl)-6-indolecarbamidine (DAPI). Coverslips were mounted onto glass slides and imaged using Nikon A1R confocal microscope.

Evaluation of cellular uptake by flow cytometry

The procedures for flow cytometry are same with our previously reported paper[1]. Specifically, HeLa cells were seeded in 24-well plates at a plating density of 1×10^6 cells per well in 1.0 mL of complete growth medium and incubated for 24 h at 37 °C in 5% CO_2 environment. Next, fresh MEM containing different samples was added to replace the original medium, and the cells without drug treatment were set as a control. The DOX concentration for free DOX, *c*(PEG-*b*-PCL)@DOX and PEG-*b*-PCL@DOX micelles in MEM was set at 49 μg/mL. After incubation for 4 h, the sample solution was aspirated, and the cells were rinsed thrice with PBS. Cells were then harvested by incubation with 200 μL of Trypsin-EDTA, followed by resuspension with 1 mL of complete growth medium. Subsequently, cells were transferred to 1.5 mL of microcentrifuge tubes and pelleted at 300g for 5 min at 4 °C. The supernatant was aspirated, and the cell pellets were resuspended in 200 μL of PBS. Cells were analyzed for uptake of fluorescent samples using a BD Accuri C6 Plus flow cytometer (BD Biosciences) with an excitation wavelength and emission wavelength of 488 nm and 595 nm, respectively. A minimum of 10,000 cells was analyzed for each sample with the fluorescence intensity.

Cell viability assay

The cytotoxicity of various formulations was evaluated *in vitro* using the 3-(4,5-dimethylthiazol-2-yl)-5-(3-carboxymethoxyphenyl)-2-(4-sulfophenyl)-2H-tetrazolium) (MTS) assay. The HeLa cells were seeded in 96-well plates at a density of 2,500 cells per well in 100 μL of complete growth medium and incubated in an incubator maintained at 37 ℃ and 5% CO_2 environment for 24 h. Free DOX, blank micelles, DOX-loaded micelles were prepared in serial dilutions in water and then diluted 10-fold in OptiMEM medium (Invitrogen). The cells were then rinsed once with PBS and incubated with 40 μL of the sample solutions at 37 °C for 24 h. Cells were then rinsed with PBS, and the medium was replaced with 100 μL of culture medium. After that, 20 μL of 3-(4,5-dimethylthiazol-2-yl)-5-(3-carbox-ymethoxyphenyl)-2-(4-sulfophenyl)-2Htetrazolium (MTS, Promega) reagent was added to each well, which were further incubated at 37 °C, 5 % CO_2 for 3 h. The absorbance of each well was measured at 490 nm on a Tecan Safire2 plate reader (Männerdorf, Switzerland). Cell viability for each treatment condition was determined by normalizing to the cells-only signal.

Degradation study

The specific steps of degradation experiment can be referred to reference 2.

References

(1) Kang, G.; Sun, L.; Liu, Y.; Meng, C.; Ma, W.; Wang, B.; Ma, L.; Yu, C.; Wei, H., Micelles with Cyclic Poly(ε-caprolactone) Moieties: Greater Stability, Larger Drug Loading Capacity, and Slower Degradation Property for Controlled Drug Release. *Langmuir* **2019**, 35 (38), 12509-12517.

(2) Wang, Y.; Wu, Z.; Ma, Z.; Tu, X.; Zhao, S.; Wang, B.; Ma, L.; Wei, H., Promotion of Micelle Stability via A Cyclic Hydrophilic Moiety. *Polym. Chem.* **2018**, *9*, 2569-2573.

Figures:

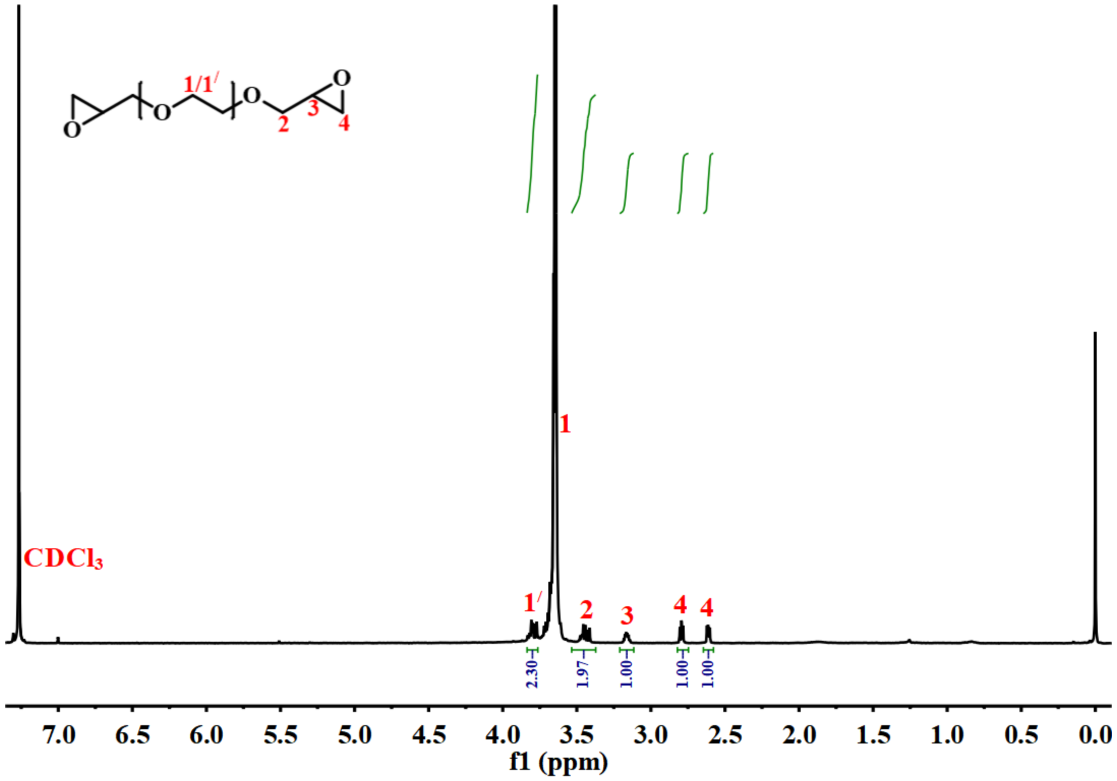

Figure S1. ^{1}H NMR spectrum of epo-PEG-epo in $CDCl_3$.

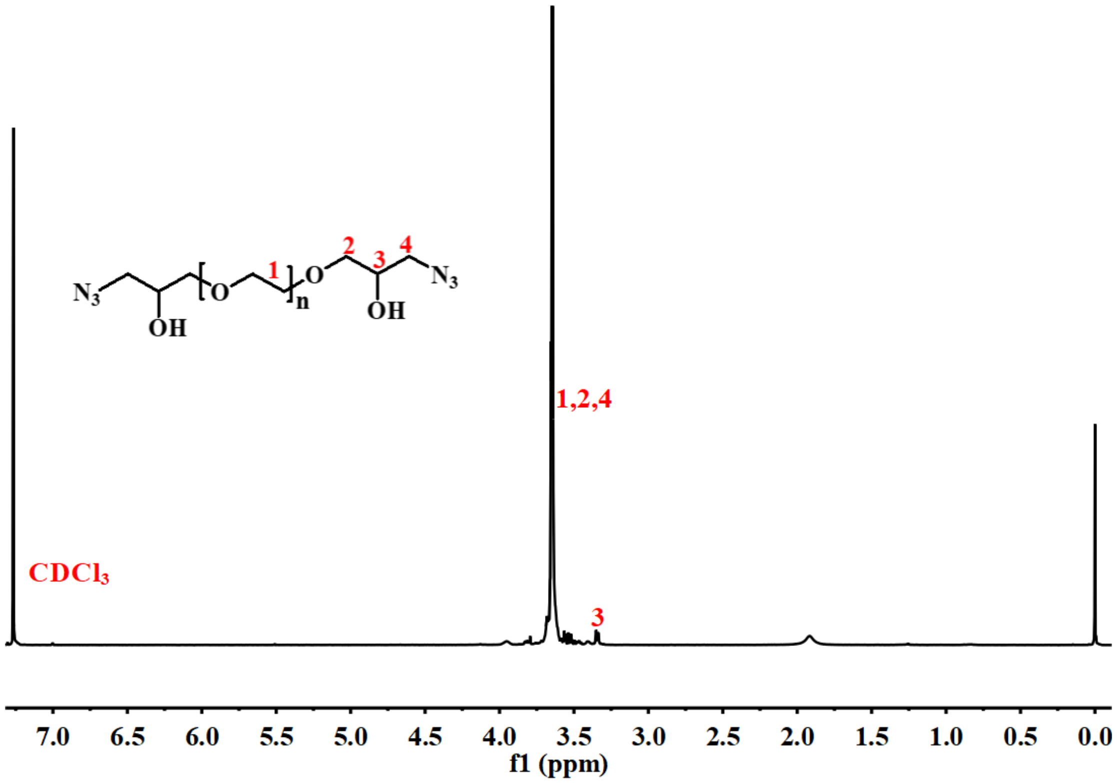

Figure S2. ^{1}H NMR spectrum of N_3-PEG-N_3 in $CDCl_3$.

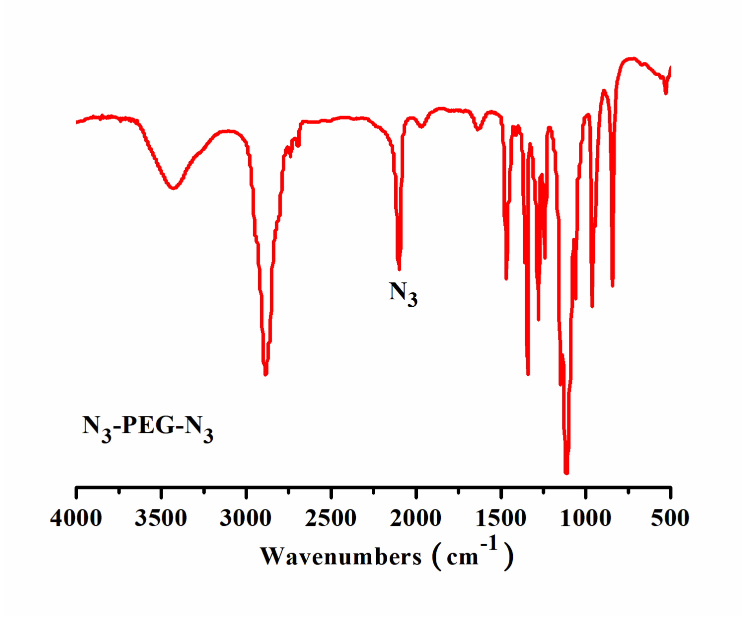

Figure S3. FT-IR spectrum of the N_3-PEG-N_3.

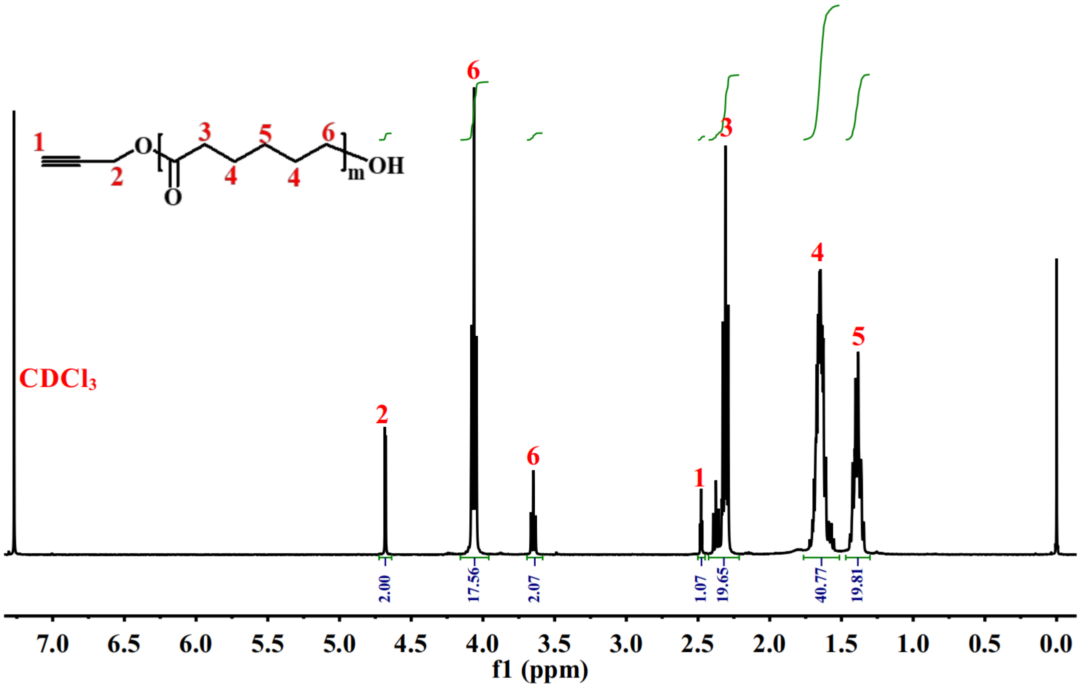

Figure S4. ^{1}H NMR spectrum of Alkynyl-PCL in $CDCl_3$.

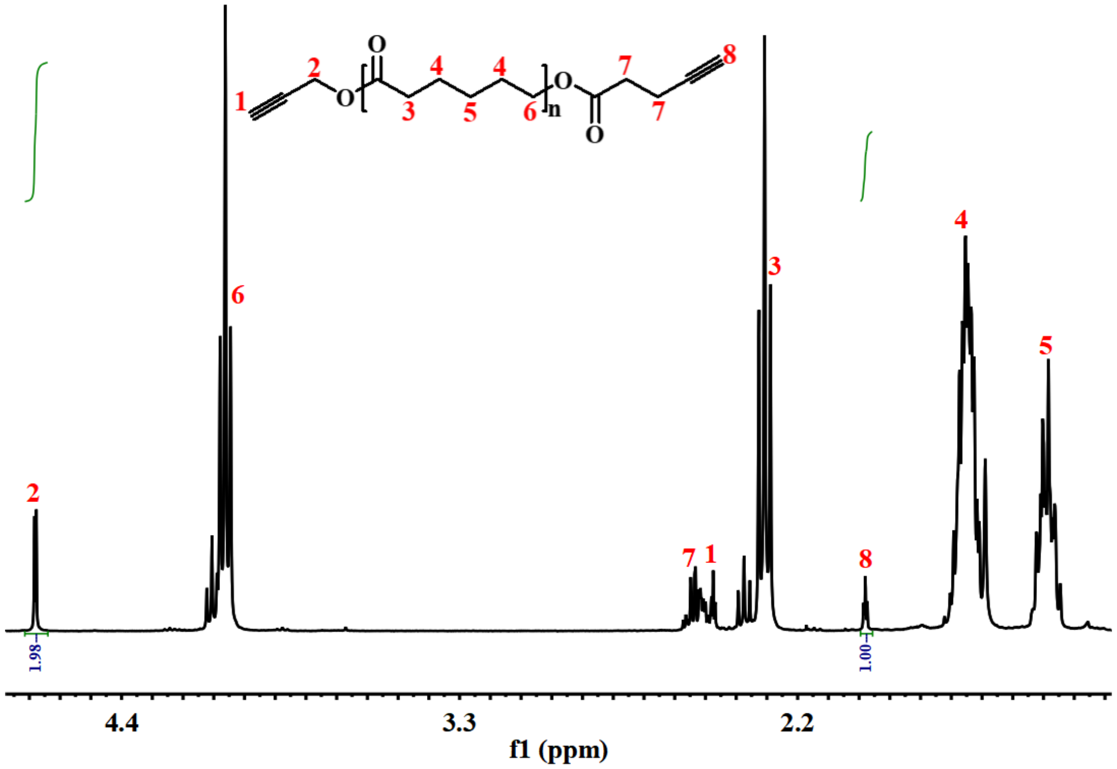

Figure S5. ^{1}H NMR spectrum of Alkynyl-PCL-Alkynyl in $CDCl_3$.

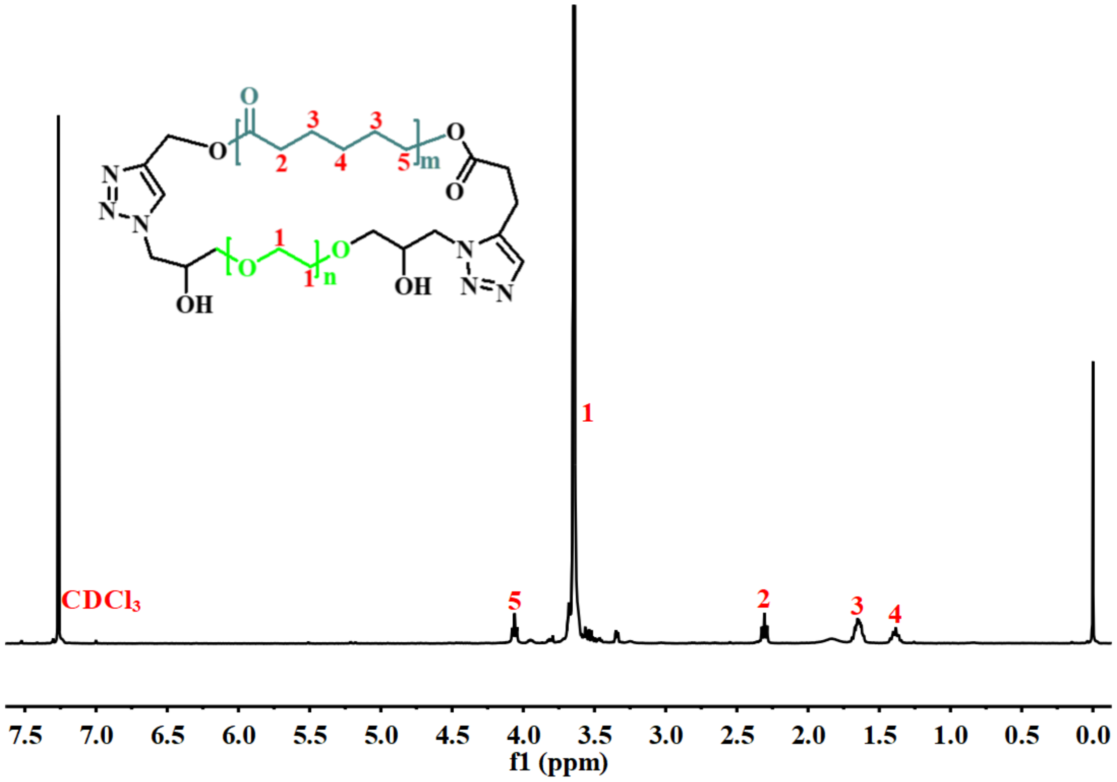

Figure S6. ^{1}H NMR spectrum of *c*(PEG-*b*-PCL) in $CDCl_3$.

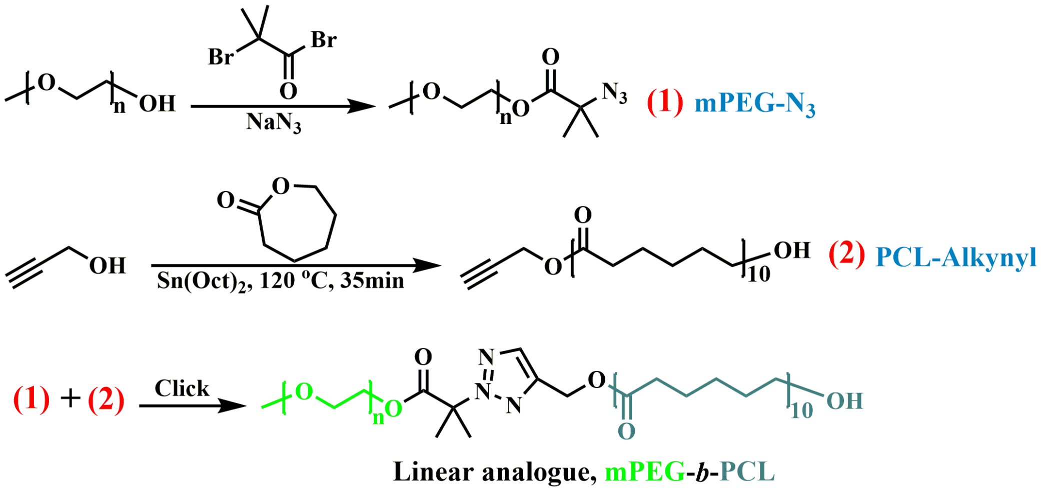

Scheme S1. Synthesis of linear analogue PEG-*b*-PCL.

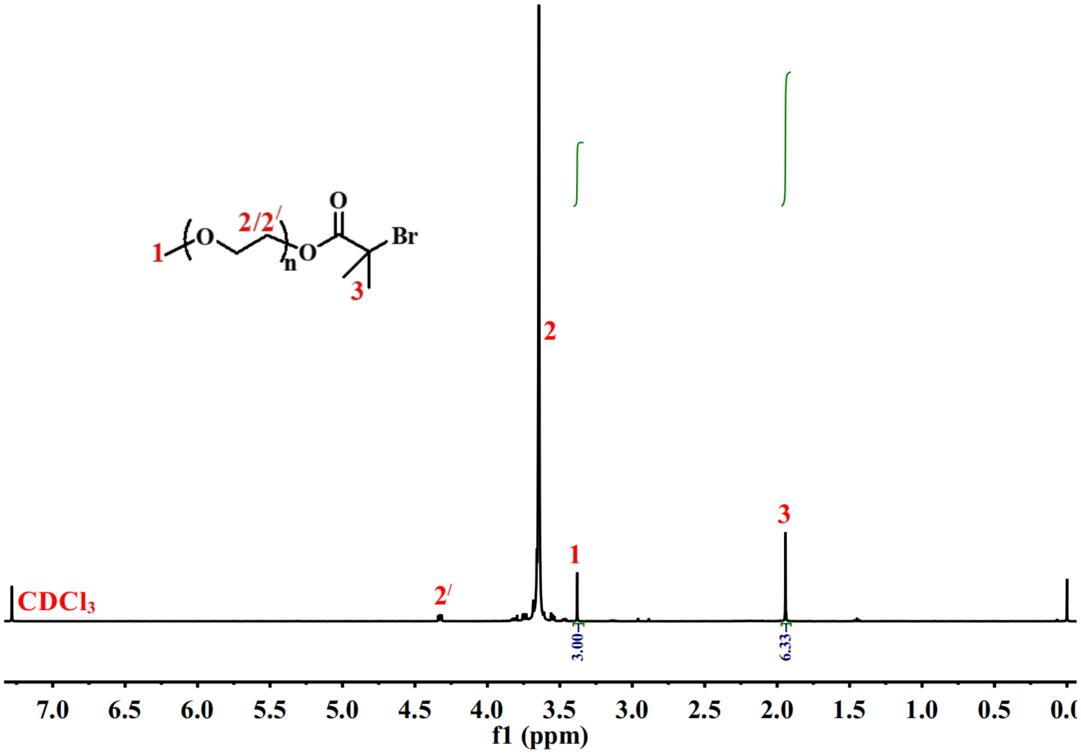

Figure S7. ^{1}H NMR spectrum of PEG-Br in $CDCl_3$.

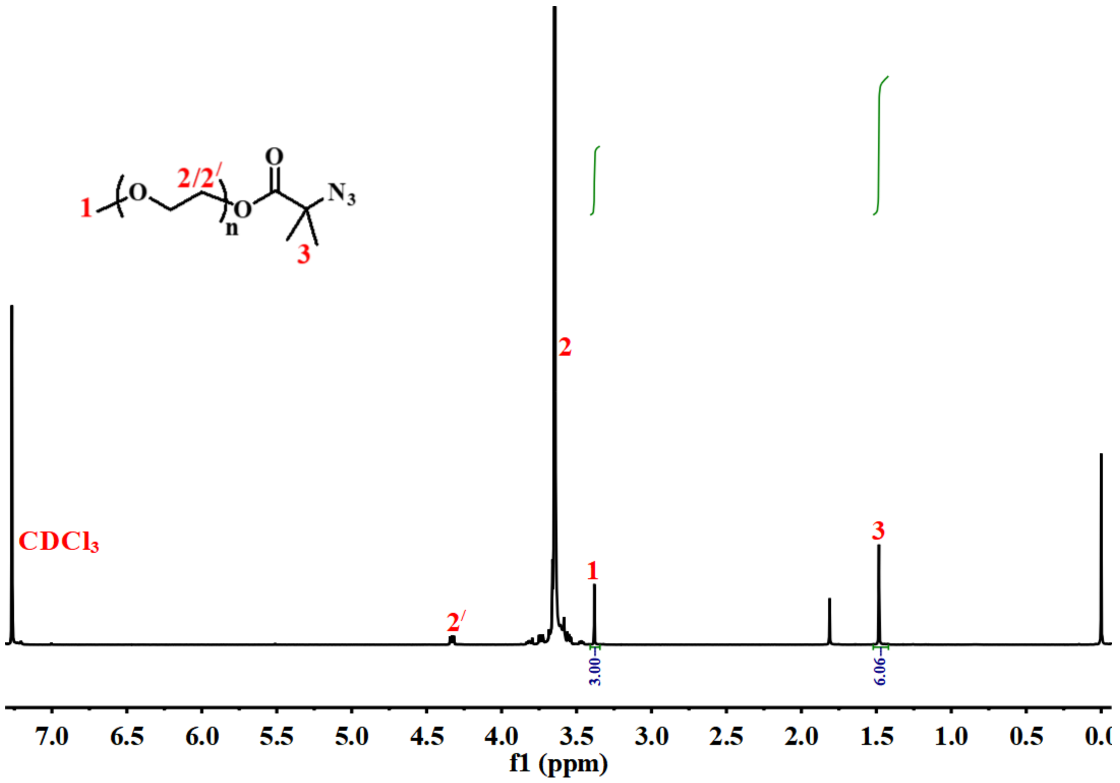

Figure S8. ^{1}H NMR spectrum of PEG-N_3 in $CDCl_3$.

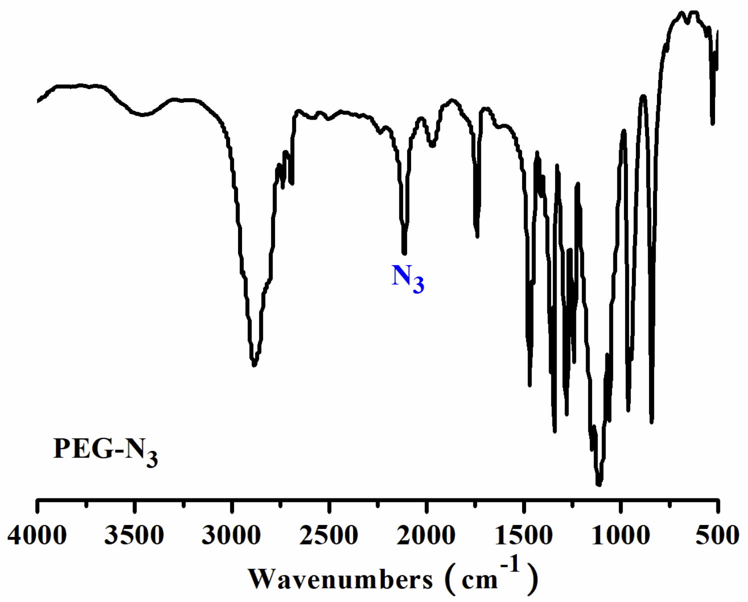

Figure S9. FT-IR spectrum of the PEG-N_3.

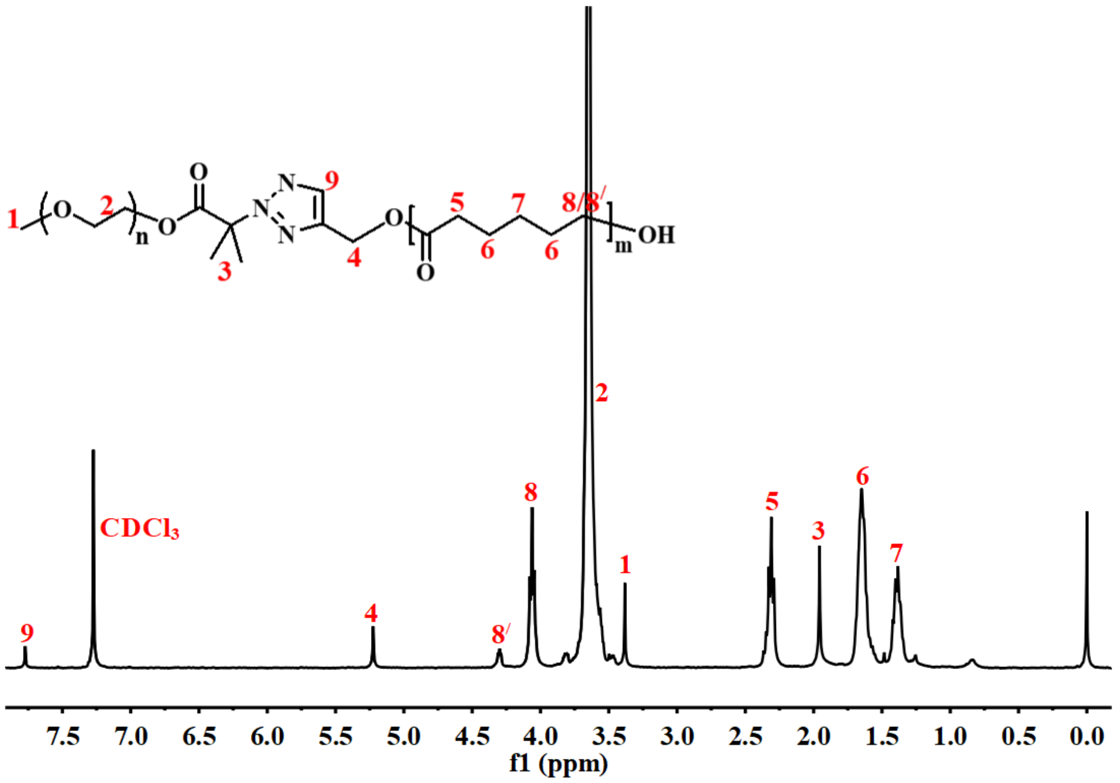

Figure S10. ^{1}H NMR spectrum of PEG-*b*-PCL in $CDCl_3$.

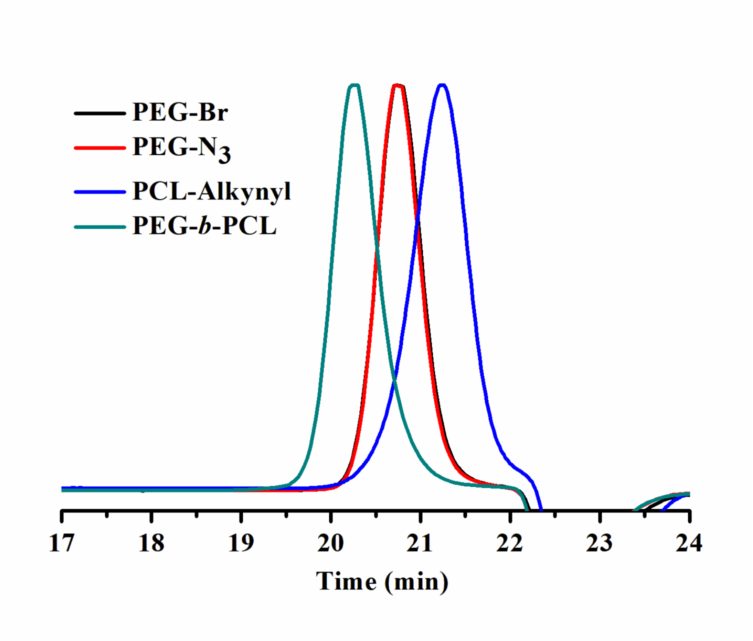

Figure S11. SEC elution traces of the PEG-Br, PEG-N_3, PCL-Alkynyl, and linear analogue PEG-*b*-PCL using DMF as an eluent.

Table S1. Summary of MW and PDI of all synthesized polymers

Samples	$M_n{}^a$ (kDa)	$M_n{}^b$ (kDa)	PDI[b]
PEG-Br	2.1	2.2	1.07
PEG-N_3	2.1	2.1	1.02
epo-PEG-epo	2.1	2.2	1.09
N_3-PEG-N_3	2.2	2.3	1.14
Alkynyl-PCL	0.8	0.8	1.15
Alkynyl-PCL-Alkynyl	0.9	0.9	1.18
PEG-*b*-PCL	2.9	3.0	1.12
c(PEG-*b*-PCL)	3.1	3.1	1.32

[a]Determined by ^{1}H NMR. [b]Determined by SEC-MALLS.

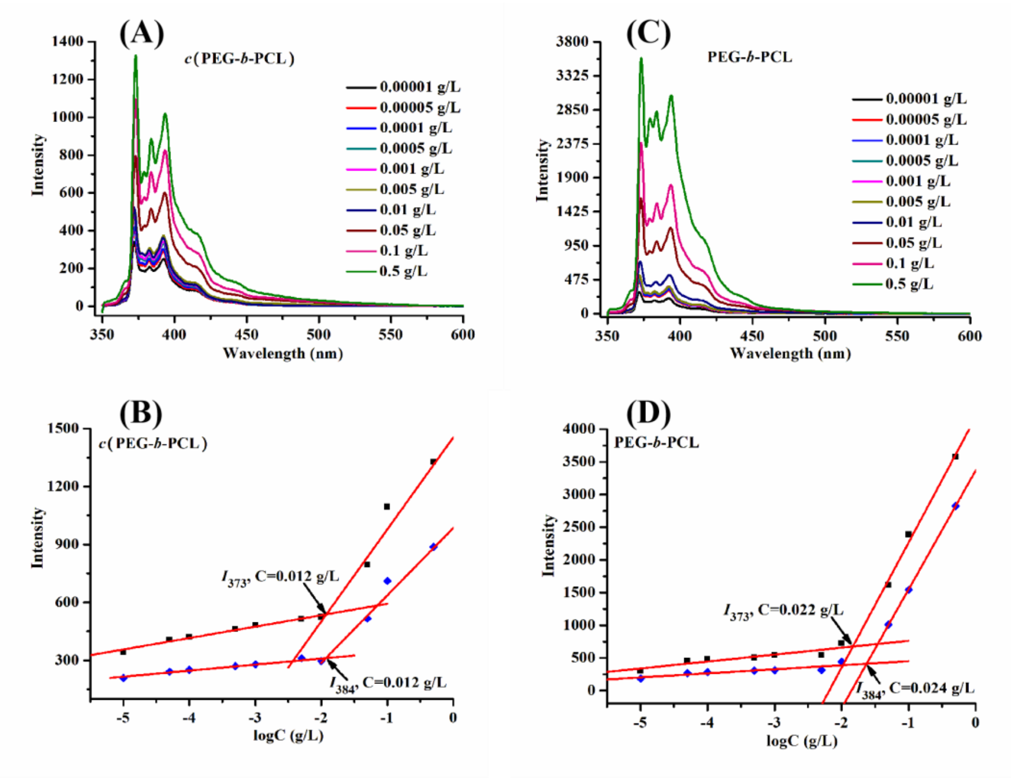

Figure S12. (A&C) Fluorescence emission spectra of pyrene at different concentrations of *c*(PEG-*b*-PCL) and PEG-*b*-PCL, (B&D) Plots of fluorescence intensity of I_{373} and I_{384} as function of logarithm of concentrations of *c*(PEG-*b*-PCL) and PEG-*b*-PCL.

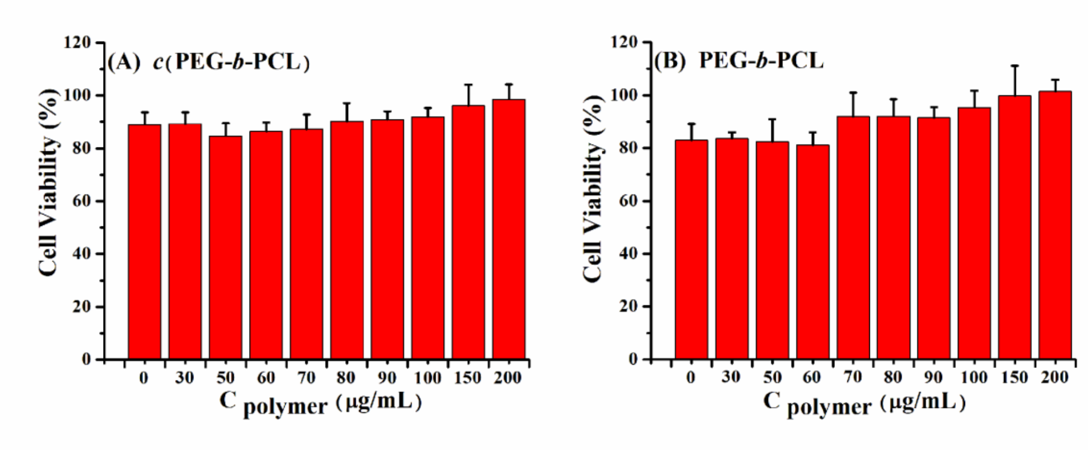

Figure S13. In vitro cytotoxicity of blank *c*(PEG-*b*-PCL) (A) and PEG-*b*-PCL micelles (B) in HeLa cells. Cell viability was determined by MTS assay and expressed as % viability compared to the untreated cells control. The data were expressed as mean ± SD, n = 3.

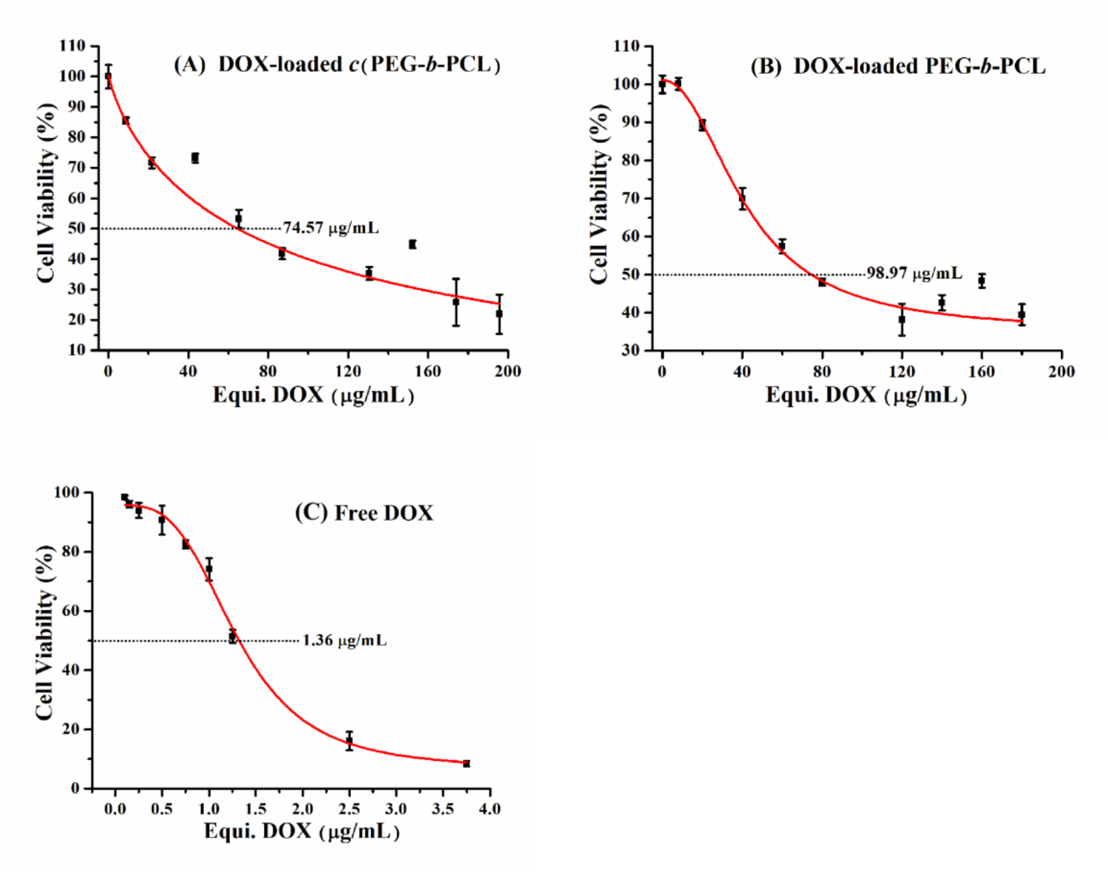

Figure S14. In vitro cytotoxicity of *c*(PEG-*b*-PCL)@DOX (A) and PEG-*b*-PCL@DOX (B) micelles and free DOX (C) in HeLa cells for 24 h of incubation. The data were expressed as mean ±SD, n = 3.

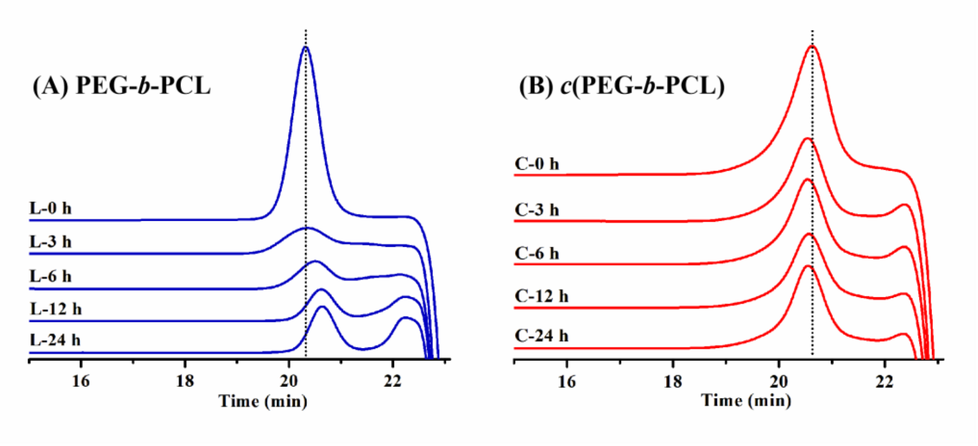

Figure S15. SEC analyses of the degraded products of linear (A) and cyclic (B) polymers at various degradation times.
